# Role of adenosine deaminase 2 gene variants in pediatric deficiency of adenosine deaminase 2: A structural biological approach

**DOI:** 10.3892/mmr.2021.12128

**Published:** 2021-05-05

**Authors:** Maria I. Zervou, George N. Goulielmos, Michail Matalliotakis, Charoula Matalliotaki, Demetrios A. Spandidos, Elias Eliopoulos

Mol Med Rep 21: 876-882, 2020; DOI: 10.3892/mmr.2019.10862

Following the publication of the above article on modeling variants of adenosine deaminase 2 (ADA2), previously identified by Gibson *et al* [Kristen M. Gibson, Kimberly A. Morishita, Paul Dancey, Paul Moorehead, Britt Drögemöller, Xiaohua Han, Jinko Graham, Robert E. W. Hancock, Dirk Foell, Susanne Benseler, Rashid Luqmani, Rae S. M.Yeung, Susan Shenoi, Marek Bohm, Alan M. Rosenberg, Colin J. Ross, David A. Cabral and Kelly L. Brown: Identification of novel adenosine deaminase 2 gene variants and varied clinical phenotype in pediatric vasculitis. Arthritis Rheumatol 71: 1747-1755, 2019], (reference 18 in the article), Dr. Kelly L. Brown, corresponding author of the Gibson *et al* article, drew to the authors' attention possible discrepancies identified therein. Upon examining the matters raised by Dr. K. Brown, the authors wish to publish a corrigendum for this article, and the following textual changes are required to the main text.

The authors noted that it was not accurate to have referred to the p.Gly47Arg mutation as being ‘novel’ when this mutation was being specifically referred to, so the word ‘novel’ should have been omitted from the sentence in the abstract starting on line 17: ‘This led to suggestions that the mutations found may affect the formation/stability of the homodimer or may influence the activity of the enzyme (15)’. However, Gibson *et al* in their paper stated that ADA2 variant with the mutation Gly47Arg in sera from homozygous individuals was a dimer (18). Also, the word ‘novel’ should not have been included in the title of Fig. 3, and this should have appeared as follows: ‘Figure 3. The DADA2-associated mutation G47R in the ADA2 structure’, and also, for consistency, the titles of Figs. 4 and 5 should have been written as ‘Figure 4. The DADA2-associated novel mutation R34W in the ADA2 structure’ and ‘Figure 5. The DADA2-associated novel mutation A357T in the ADA2 structure’. The authors would like to add that the p.Arg34Trp variant's association with DADA2 has been previously identified in a paper by Kaljas *et al*: Human adenosine deaminases ADA1 and ADA2 bind to different subsets of immune cells. Kaijas Y, Liu C, Skaldin M, Wu C, Zhou Q, Lu Y, Aksentijevich I and Zavialow AV: Cell Mol Life Sci 74: 550-570, 2017.

In addition, the novel rare mutation identified by Gibson *et al* as Arg9Trp associated with DADA2 lies in the signal peptide [stated by the authors as Arg8Trp because Met#1 (ATG start codon) is not included in the protein numbering] and is not obviously included in the three-dimensional structure, and therefore the authors did not deal with it. So, the sentence in the Abstract starting on line 19 should have been written as follows: ‘It was thus concluded that the Gly47Arg mutation affects the position and interaction of the dimer-associated HN1 helical structure’. All references of Arg8Trp in the text, when referred to the Gibson *et al* article, should be changed to Arg9Trp as referred therein so as not to cause confusion.

Finally, in the legend for Fig. 2, ‘His358’ should have been written as ‘His356’ (line 3), and for the purposes of clarification, where ‘at the next Asn352 (2)’ was written at the end of the same sentence, this text should be changed to ‘at the neighboring glycosylated Asn378 [Asn352 in (2)]’. Similarly, on p. 880, the sentence at the end of the penultimate paragraph of the Results section should have been written as follows: ‘This disruption could be transmitted to the neighboring His356 coordinated to the metal ion or affect the confirmed glycosylation at the neighboring glycosylated Asn378 [Asn352 in (2)]’.

The authors thank Dr. K. L. Brown for drawing these matters to their attention, and emphasize that the resultant corrections and clarifications do not alter either the results or the main conclusions reported in the paper.

